# Supplementary material for: A Systematic Review of the Current Measures of Theory of Mind in Adults with Schizophrenia
Source: Int J Environ Res Public Health. 2021 Jul 4;18(13):7172. doi: 10.3390/ijerph18137172 (PMC8297277; doi:10.3390/ijerph18137172)
Supplement: Supplementary file 1 [file ijerph-18-07172-s001.zip › ijerph-1219266-SI.pdf]

Table S1. Quality assessment of the studies included for the evaluation of psychometric properties.

Theory of mind task: Hinting

COSMIN Box	Quality assessment						
Reference	Item 1	Item 2	Item 3	Item 4	Item 5	Item 6	Item 7
Box 4: Internal consistency							
Lindgren (2018)	D	V	NA	NA	D	--	--
Box 6: Reliability							
Pinkham (2006)	A	D	A	D	NA	--	--
Box 9a: Convergent validity							
Achim Ouellet, Roy & Jackson (2012)	V	V	V	V	--	--	--
Bora et al., (2008)	V	D	V	V	--	--	--
Corcoran & Frith (2003)	V	D	V	A	--	--	--
Mehl et al., (2010a)	A	A	D	A	--	--	--
Mehl et al., (2010b)	V	V	V	V	--	--	--
Box 9b: discriminative or known-group validity							
Achim Ouellet, Roy & Jackson (2012)	--	--	--	--	V	V	V
Bora et al., (2008)	--	--	--	--	V	V	V
Bozikas (2011)	--	--	--	--	V	V	V
Corcoran & Frith (2003)	--	--	--	--	V	V	V
Corcoran, Mercer & Frith (1995)	--	--	--	--	V	V	V
Lindgren (2018)	--	--	--	--	V	D	D
Marjoram (2005)	--	--	--	--	V	V	V
Mehl et al., (2010a)	--	--	--	--	V	V	V
Mehl et al., (2010b)	--	--	--	--	V	V	V
Pinkham (2006)	--	--	--	--	V	V	V
Thompson (2012)	--	--	--	--	V	V	V

Note: V = very good; A = adequate; D = doubtful; I = inadequate; NA = not applicable.
Box 4. Item 1 = Was an internal consistency statistic calculated for each unidimensional scale or subscale separately?
Box 4. Item 2 = For continuous scores: Was Cronbach's alpha or omega calculated?
Box 4. Item 3 = For dichotomous scores: Was Cronbach's alpha or KR- 20 calculated?
Box 4. Item 4 = For IRT-based scores: Was standard error of the theta (SE (θ)) or reliability coefficient of estimated latent trait value (index of (subject or item) separation) calculated?
Box 4. Item 5 = Were there any other important flaws in the design or statistical methods of the study?
Box 6. Item 1 = Were patients stable in the interim period on the construct to be measured?
Box 6. Item 2 = Was the time interval appropriate?
Box 6. Item 3 = Were the test conditions similar for the measurements? e.g. type of administration, environment, instructions
Box 6. Item 4 = For continuous scores: Was an intraclass correlation coefficient (ICC) calculated?
Box 6. Item 5 = For dichotomous/nominal/ordinal scores: Was kappa calculated?
Box 9a. Item 1 = Is it clear what the comparator instrument(s) measure(s)?
Box 9a. Item 2 = Were the measurement properties of the comparator instrument(s) sufficient?
Box 9a. Item 3 = Was the statistical method appropriate for the hypotheses to be tested?
Box 9a. Item 4 = Were there any other important flaws in the design or statistical methods of the study?
Box 9b. Item 5 = Was an adequate description provided of important characteristics of the subgroups?
Box 9b. Item 6 = Was the statistical method appropriate for the hypotheses to be tested?
Box 9b. Item 7 = Were there any other important flaws in the design or statistical methods of the study?

Theory of mind task: first-order False Belief

COSMIN Box	Quality assessment						
Reference	Item 1	Item 2	Item 3	Item 4	Item 5	Item 6	Item 7
Box 6: Reliability							
Pinkham & Penn (2006)	A	D	A	NA	D	--	--
Box 9b: discriminative or known-group validity							
Bâ (2008)	–	–	–	–	V	V	V
Bozikas (2011)	–	–	–	–	V	V	V
Brüne (2001)	–	–	–	–	V	V	V
Brüne (2005)	–	–	–	–	V	V	V
Doddy et al., (1998)	–	–	–	–	V	V	V
Frith & Corcoran (1996)	–	–	–	–	V	V	V
Herold (2002)	–	–	–	–	V	D	D
Hur (2013)	–	–	–	–	V	V	V
Jassen (2003)	–	–	–	–	V	I	D
Langdon, Connors and Connaughton (2017)	–	–	–	–	V	V	V
Mazza (2001)	–	–	–	–	V	V	V
Pentaraki (2012)	–	–	–	–	V	V	V
Pickup & Frith (2001)	–	–	–	–	V	V	V
Pinkham (2006)	–	–	–	–	V	D	D
Randell et al., (2003)	–	–	–	–	V	V	V

Note: V = very good; A = adequate; D = doubtful; I = inadequate; NA = not applicable.
Box 6. Item 1 = Were patients stable in the interim period on the construct to be measured?
Box 6. Item 2 = Was the time interval appropriate?
Box 6. Item 3 = Were the test conditions similar for the measurements? e.g. type of administration, environment, instructions
Box 6. Item 4 = For continuous scores: Was an intraclass correlation coefficient (ICC) calculated?
Box 6. Item 5 = For dichotomous/nominal/ordinal scores: Was kappa calculated?
Box 9b. Item 5 = Was an adequate description provided of important characteristics of the subgroups?
Box 9b. Item 6 = Was the statistical method appropriate for the hypotheses to be tested?
Box 9b. Item 7 = Were there any other important flaws in the design or statistical methods of the study?

Theory of mind task: second-order False Belief

COSMIN Box	Quality assessment						
Reference	Item 1	Item 2	Item 3	Item 4	Item 5	Item 6	Item 7
Box 6: Reliability							
Pinkham & Penn (2006)	A	D	A	NA	D	--	--
Box 9b: discriminative or known-group validity							
Bâ (2008)	–	–	–	–	V	V	V
Bozikas (2011)	–	–	–	–	V	V	V
Brüne (2001)	–	–	–	–	V	V	V
Brüne (2005)	–	–	–	–	V	V	V
Doddy et al., (1998)	–	–	–	–	V	V	V
Frith & Corcoran (1996)	–	–	–	–	V	V	V
Herold (2002)	–	–	–	–	V	D	D
Hur (2013)	–	–	–	–	V	V	V
Langdon, Connors and Connaughton (2017)	–	–	–	–	V	V	V
Mazza (2001)	–	–	–	–	V	V	V
Pentaraki (2012)	–	–	–	–	V	V	V
Pickup & Frith (2001)	–	–	–	–	V	V	V
Pinkham (2006)	–	–	–	–	V	D	D
Randell et al., (2003)	–	–	–	–	V	V	V

Note: V = very good; A = adequate; D = doubtful; I = inadequate; NA = not applicable.
Box 6. Item 1 = Were patients stable in the interim period on the construct to be measured?
Box 6. Item 2 = Was the time interval appropriate?
Box 6. Item 3 = Were the test conditions similar for the measurements? e.g. type of administration, environment, instructions
Box 6. Item 4 = For continuous scores: Was an intraclass correlation coefficient (ICC) calculated?
Box 6. Item 5 = For dichotomous/nominal/ordinal scores: Was kappa calculated?
Box 9b. Item 5 = Was an adequate description provided of important characteristics of the subgroups?
Box 9b. Item 6 = Was the statistical method appropriate for the hypotheses to be tested?
Box 9b. Item 7 = Were there any other important flaws in the design or statistical methods of the study?

Theory of mind task: False Belief picture sequencing

COSMIN Box	Quality assessment						
Reference	Item 1	Item 2	Item 3	Item 4	Item 5	Item 6	Item 7
Box 3: Structural validity							
Corcoran et al., (2011)	V	D	A	--	--	--	--
Box 4: Internal consistency							
Faisca et al., (2016)	V	V	NA	NA	A	--	--
Box 9b: discriminative or known-group validity							
Anselmetti et al., (2009)	--	--	--	--	V	V	V
Brüne (2003)	--	--	--	--	V	V	V
Brüne (2005)	--	--	--	--	V	V	V
Brüne et al., (2008)	--	--	--	--	V	V	V
Brüne et al., (2011)	--	--	--	--	V	D	A
Langdon (1997)	--	--	--	--	V	V	V
Langdon (2001)	--	--	--	--	V	V	V
Langdon (2002)	--	--	--	--	V	V	V
Langdon, Coltheart & Ward (2006)	--	--	--	--	V	V	V
Langdon et al., (2014)	--	--	--	--	V	V	V
Langdon, Connors & Connaughton (2017)	--	--	--	--	V	V	V
Zalla (2006)	--	--	--	--	V	V	V

Note: V = very good; A = adequate; D = doubtful; I = inadequate; NA = not applicable.
Box 3. Item 1 = For CTT: Was exploratory or confirmatory factor analysis performed?
Box 3. Item 2 = For IRT/Rasch: does the chosen model fit to the research question?
Box 3. Item 3 = Was the sample size included in the analysis adequate?
Box 4. Item 1 = Was an internal consistency statistic calculated for each unidimensional scale or subscale separately?
Box 4. Item 2 = For continuous scores: Was Cronbach's alpha or omega calculated?
Box 4. Item 3 = For dichotomous scores: Was Cronbach's alpha or KR- 20 calculated?
Box 4. Item 4 = For IRT-based scores: Was standard error of the theta (SE (θ)) or reliability coefficient of estimated latent trait value (index of (subject or item) separation) calculated?
Box 4. Item 5 = Were there any other important flaws in the design or statistical methods of the study?
Box 9b. Item 5 = Was an adequate description provided of important characteristics of the subgroups?
Box 9b. Item 6 = Was the statistical method appropriate for the hypotheses to be tested?
Box 9b. Item 7 = Were there any other important flaws in the design or statistical methods of the study?

Theory of mind task: Character Intention task

COSMIN Box	Quality assessment						
Reference	Item 1	Item 2	Item 3	Item 4	Item 5	Item 6	Item 7
Box 9b: discriminative or known-group validity							
Brunet (2003)	—	—	—	—	V	V	V
Brunet et al., (2003)	—	—	—	—	V	V	V
Hur et al., (2013)	—	—	—	—	V	V	V
Sarfati (1997)	—	—	—	—	V	V	V

Note: V = very good; A = adequate; D = doubtful; I = inadequate; NA = not applicable.
Box 9b. Item 5 = Was an adequate description provided of important characteristics of the subgroups?
Box 9b. Item 6 = Was the statistical method appropriate for the hypotheses to be tested?
Box 9b. Item 7 = Were there any other important flaws in the design or statistical methods of the study?

Theory of mind task: Visual Jokes

COSMIN Box	Quality assessment						
Reference	Item 1	Item 2	Item 3	Item 4	Item 5	Item 6	Item 7
Box 4: Internal consistency							
Polimeni et al., (2010)	V	V	NA	NA	A	--	--
Box 9b: discriminative or known-group validity							
Corcoran (1997)	--	--	--	--	V	V	V
Langdon, Connors & Connaughton (2017)	--	--	--	--	V	V	V
Marjoram et al., (2005)	--	--	--	--	V	V	V
Polimeni et al., (2010)	--	--	--	--	V	V	V
Thompson et al., (2012)	--	--	--	--	V	V	V

Note: V = very good; A = adequate; D = doubtful; I = inadequate; NA = not applicable.
Box 4. Item 1 = Was an internal consistency statistic calculated for each unidimensional scale or subscale separately?
Box 4. Item 2 = For continuous scores: Was Cronbach's alpha or omega calculated?
Box 4. Item 3 = For dichotomous scores: Was Cronbach's alpha or KR- 20 calculated?
Box 4. Item 4 = For IRT-based scores: Was standard error of the theta (SE (θ)) or reliability coefficient of estimated latent trait value (index of (subject or item) separation) calculated?
Box 4. Item 5 = Were there any other important flaws in the design or statistical methods of the study?
Box 9b. Item 5 = Was an adequate description provided of important characteristics of the subgroups?
Box 9b. Item 6 = Was the statistical method appropriate for the hypotheses to be tested?
Box 9b. Item 7 = Were there any other important flaws in the design or statistical methods of the study?

Theory of mind task: Irony

COSMIN Box	Quality assessment						
Reference	Item 1	Item 2	Item 3	Item 4	Item 5	Item 6	Item 7
Box 9b: discriminative or known-group validity							
Champagne-Lavau et al., (2012)	—	—	—	—	V	V	V
Herold (2002)	—	—	—	—	V	D	A
Langdon, Davies, Martin & Max (2002)	—	—	—	—	V	V	V
Mitchley et al., (1998)	—	—	—	—	V	V	V
Rapp et al., (2013)	—	—	—	—	V	D	A

Note: V = very good; A = adequate; D = doubtful; I = inadequate; NA = not applicable.
Box 9b. Item 5 = Was an adequate description provided of important characteristics of the subgroups?
Box 9b. Item 6 = Was the statistical method appropriate for the hypotheses to be tested?
Box 9b. Item 7 = Were there any other important flaws in the design or statistical methods of the study?

Theory of mind task: Faux Pas

COSMIN Box	Quality assessment						
Reference	Item 1	Item 2	Item 3	Item 4	Item 5	Item 6	Item 7
Box 4: Internal consistency							
Faisca et al., (2016)	NA	V	NA	NA	V	--	--
Box 6: Reliability							
Chen et al., (2017)	A	V	V	NA	V	--	--
Box 9a: Convergent validity							
Martino et al., (2007)	V	V	V	V	--	--	--
Shur, Shamay-Tsoory & Levkovitz (2008)	V	V	D	D	--	--	--
Zhu et al., (2007)	V	V	V	V	--	--	--
Box 9b: discriminative or known-group validity							
de Achával et al., (2010)	--	--	--	--	V	V	V
Faisca et al., (2016)	--	--	--	--	V	V	v
Herold et al., (2009)	--	--	--	--	V	V	V
Ho et al., (2015)	--	--	--	--	V	V	V
Hooker et al., (2011)	--	--	--	--	V	V	V
Li et al., (2017)	--	--	--	--	V	V	V
Martino et al., (2007)	--	--	--	--	V	V	V
Shur, Shamay-Tsoory & Levkovitz (2008)	--	--	--	--	V	V	V
Zhu et al., (2007)	--	--	--	--	V	V	V

Note: V = very good; A = adequate; D = doubtful; I = inadequate; NA = not applicable.
Box 4. Item 1 = Was an internal consistency statistic calculated for each unidimensional scale or subscale separately?
Box 4. Item 2 = For continuous scores: Was Cronbach's alpha or omega calculated?
Box 4. Item 3 = For dichotomous scores: Was Cronbach's alpha or KR- 20 calculated?
Box 4. Item 4 = For IRT-based scores: Was standard error of the theta (SE (θ)) or reliability coefficient of estimated latent trait value (index of (subject or item) separation) calculated?
Box 4. Item 5 = Were there any other important flaws in the design or statistical methods of the study?
Box 6. Item 1 = Were patients stable in the interim period on the construct to be measured?
Box 6. Item 2 = Was the time interval appropriate?
Box 6. Item 3 = Were the test conditions similar for the measurements? e.g. type of administration, environment, instructions
Box 6. Item 4 = For continuous scores: Was an intraclass correlation coefficient (ICC) calculated?
Box 6. Item 5 = For dichotomous/nominal/ordinal scores: Was kappa calculated?
Box 9a. Item 1 = Is it clear what the comparator instrument(s) measure(s)?
Box 9a. Item 2 = Were the measurement properties of the comparator instrument(s) sufficient?
Box 9a. Item 3 = Was the statistical method appropriate for the hypotheses to be tested?
Box 9a. Item 4 = Were there any other important flaws in the design or statistical methods of the study?
Box 9b. Item 5 = Was an adequate description provided of important characteristics of the subgroups?
Box 9b. Item 6 = Was the statistical method appropriate for the hypotheses to be tested?
Box 9b. Item 7 = Were there any other important flaws in the design or statistical methods of the study?

Theory of mind task: Yoni's Verbal and Eye Gaze Cues

COSMIN Box	Quality assessment						
Reference	Item 1	Item 2	Item 3	Item 4	Item 5	Item 6	Item 7
Box 9b: discriminative or known-group validity							
Ho et al., (2015)	—	—	—	—	V	V	V
Ho et al., (2018)	—	—	—	—	V	V	V
Li et al., (2017)	—	—	—	—	V	V	V
Shamay-Tsoory, Aharon-Peretz & Levkovitz (2007)	—	—	—	—	V	V	V
Shamay-Tsoory et ai., (2007)	—	—	—	—	V	V	V
Wang et al., (2017)	—	—	—	—	V	V	V

Note: V = very good; A = adequate; D = doubtful; I = inadequate; NA = not applicable.
Box 9b. Item 5 = Was an adequate description provided of important characteristics of the subgroups?
Box 9b. Item 6 = Was the statistical method appropriate for the hypotheses to be tested?
Box 9b. Item 7 = Were there any other important flaws in the design or statistical methods of the study?

Theory of mind task: Story test

COSMIN Box	Quality assessment						
Reference	Item 1	Item 2	Item 3	Item 4	Item 5	Item 6	Item 7
Box 6: Reliability							
Chen et al., (2017)	A	D	A	NA	D	--	--
Box 9b: discriminative or known-group validity							
de Achával et al., (2010)	—	—	—	—	V	V	V
Hur et al., (2013)	—	—	—	—	V	V	V
Okruszek et al., (2018)	—	—	—	—	V	V	V
Rossell & Van Rheenen (2013)	—	—	—	—	V	V	V

Note: V = very good; A = adequate; D = doubtful; I = inadequate; NA = not applicable.
Box 6. Item 1 = Were patients stable in the interim period on the construct to be measured?
Box 6. Item 2 = Was the time interval appropriate?
Box 6. Item 3 = Were the test conditions similar for the measurements? e.g. type of administration, environment, instructions
Box 6. Item 4 = For continuous scores: Was an intraclass correlation coefficient (ICC) calculated?
Box 6. Item 5 = For dichotomous/nominal/ordinal scores: Was kappa calculated?
Box 9b. Item 5 = Was an adequate description provided of important characteristics of the subgroups?
Box 9b. Item 6 = Was the statistical method appropriate for the hypotheses to be tested?
Box 9b. Item 7 = Were there any other important flaws in the design or statistical methods of the study?

Theory of mind task: Movie for Social Cognition

COSMIN Box	Quality assessment						
Reference	Item 1	Item 2	Item 3	Item 4	Item 5	Item 6	Item 7
Box 9a: Convergent validity							
Canty, Neuman & Shum (2017)	V	V	V	V	--	--	--
Montag et al., (2011)	V	V	V	V	--	--	--
Vaskinn et al., (2018)	V	V	V	V	--	--	--
Box 9b: discriminative or known-group validity							
Canty, Neuman & Shum (2017)	--	--	--	--	V	V	V
Montag et al., (2011)	--	--	--	--	V	V	V
Vaskinn et al., (2018)	--	--	--	--	V	V	V

Note: V = very good; A = adequate; D = doubtful; I = inadequate; NA = not applicable.
Box 9a. Item 1 = Is it clear what the comparator instrument(s) measure(s)?
Box 9a. Item 2 = Were the measurement properties of the comparator instrument(s) sufficient?
Box 9a. Item 3 = Was the statistical method appropriate for the hypotheses to be tested?
Box 9a. Item 4 = Were there any other important flaws in the design or statistical methods of the study?
Box 9b. Item 5 = Was an adequate description provided of important characteristics of the subgroups?
Box 9b. Item 6 = Was the statistical method appropriate for the hypotheses to be tested?
Box 9b. Item 7 = Were there any other important flaws in the design or statistical methods of the study?

Theory of mind task: Reading the Mind in the Eyes Test

COSMIN Box	Quality assessment						
Reference	Item 1	Item 2	Item 3	Item 4	Item 5	Item 6	Item 7
Box 4: Internal consistency							
Pinkham et al., (2016)	A	V	NA	NA	V	--	--
Box 6: Reliability							
Chen et al., (2017)	A	V	V	V	NA	--	--
Pinkham et al., (2016)	A	V	V	V	NA	--	--
Box 9a: Convergent validity							
Bora et al., (2006)	V	V	V	V	--	--	--
Box 9b: discriminative or known-group validity							
Ayesa-Arriola et al., (2016)	--	--	--	--	V	V	V
Bora et al., (2006)	--	--	--	--	V	V	V
Couture et al., (2008)	--	--	--	--	V	V	V
de Achával et al., (2010)	--	--	--	--	V	V	V
Kelemen et al., (2005)	--	--	--	--	V	V	V
Pentaraki et al., (2012)	--	--	--	--	V	V	V
Pinkham et al., (2016)	--	--	--	--	V	V	V
Shur, Shamay-Tsoory & Levkovitz (2008)	--	--	--	--	V	V	V

Note: V = very good; A = adequate; D = doubtful; I = inadequate; NA = not applicable.
Box 4. Item 1 = Was an internal consistency statistic calculated for each unidimensional scale or subscale separately?
Box 4. Item 2 = For continuous scores: Was Cronbach’s alpha or omega calculated?
Box 4. Item 3 = For dichotomous scores: Was Cronbach’s alpha or KR- 20 calculated?
Box 4. Item 4 = For IRT-based scores: Was standard error of the theta (SE (θ)) or reliability coefficient of estimated latent trait value (index of (subject or item) separation) calculated?
Box 4. Item 5 = Were there any other important flaws in the design or statistical methods of the study?
Box 6. Item 1 = Were patients stable in the interim period on the construct to be measured?
Box 6. Item 2 = Was the time interval appropriate?
Box 6. Item 3 = Were the test conditions similar for the measurements? e.g. type of administration, environment, instructions
Box 6. Item 4 = For continuous scores: Was an intraclass correlation coefficient (ICC) calculated?
Box 6. Item 5 = For dichotomous/nominal/ordinal scores: Was kappa calculated?
Box 9a. Item 1 = Is it clear what the comparator instrument(s) measure(s)?
Box 9a. Item 2 = Were the measurement properties of the comparator instrument(s) sufficient?
Box 9a. Item 3 = Was the statistical method appropriate for the hypotheses to be tested?
Box 9a. Item 4 = Were there any other important flaws in the design or statistical methods of the study?
Box 9b. Item 5 = Was an adequate description provided of important characteristics of the subgroups?
Box 9b. Item 6 = Was the statistical method appropriate for the hypotheses to be tested?
Box 9b. Item 7 = Were there any other important flaws in the design or statistical methods of the study?

Theory of mind task: Moving Shapes

COSMIN Box	Quality assessment						
Reference	Item 1	Item 2	Item 3	Item 4	Item 5	Item 6	Item 7
Box 3: Structural validity							
Lee et al., (2018)	V	D	A	--	--	--	--
Box 4: Internal consistency							
Bell et al., (2010)	A	V	NA	NA	V	--	--
Lee et al., (2018)							
Box 9a: Convergent validity							
Bell et al., (2010)	V	V	V	V	--	--	--
Ventura et al., (2015)	V	V	V	V	--	--	--
Ventura et al., (2013)	V	V	V	V	--	--	--
Box 9b: discriminative or known-group validity							
Bell et al., (2010)	--	--	--	--	V	V	V
Das et al., (2012)	--	--	--	--	V	V	V
Koelkebeck et al., (2010)	--	--	--	--	V	V	V
Lee et al., (2018)	--	--	--	--	V	V	V
Ventura et al., (2015)	--	--	--	--	V	V	V

Note: V = very good; A = adequate; D = doubtful; I = inadequate; NA = not applicable.
Box 3. Item 1 = For CTT: Was exploratory or confirmatory factor analysis performed?
Box 3. Item 2 = For IRT/Rasch: does the chosen model fit to the research question?
Box 3. Item 3 = Was the sample size included in the analysis adequate?
Box 4. Item 1 = Was an internal consistency statistic calculated for each unidimensional scale or subscale separately?
Box 4. Item 2 = For continuous scores: Was Cronbach's alpha or omega calculated?
Box 4. Item 3 = For dichotomous scores: Was Cronbach's alpha or KR- 20 calculated?
Box 4. Item 4 = For IRT-based scores: Was standard error of the theta (SE (θ)) or reliability coefficient of estimated latent trait value (index of (subject or item) separation) calculated?
Box 4. Item 5 = Were there any other important flaws in the design or statistical methods of the study?
Box 9a. Item 1 = Is it clear what the comparator instrument(s) measure(s)?
Box 9a. Item 2 = Were the measurement properties of the comparator instrument(s) sufficient?
Box 9a. Item 3 = Was the statistical method appropriate for the hypotheses to be tested?
Box 9a. Item 4 = Were there any other important flaws in the design or statistical methods of the study?
Box 9b. Item 5 = Was an adequate description provided of important characteristics of the subgroups?
Box 9b. Item 6 = Was the statistical method appropriate for the hypotheses to be tested?
Box 9b. Item 7 = Were there any other important flaws in the design or statistical methods of the study?
